# Supplementary material for: Stable machine-learning parameterization of subgrid processes for climate modeling at a range of resolutions
Source: Nat Commun. 2020 Jul 3;11:3295. doi: 10.1038/s41467-020-17142-3 (PMC7335176; doi:10.1038/s41467-020-17142-3)
Supplement: Supplementary file 1 — Supplementary Information [file 41467_2020_17142_MOESM1_ESM.pdf]

Supplementary Information
Stable machine-learning parameterization of subgrid processes for climate modeling at a range of resolutions

Yuval and O'Gorman

Supplementary Note 1

Here we show that the RF parameterization performs well in respecting physical constraints. This good performance arises because the constraints are respected by the training data and the RF predictions are averages over subsets of the training data[1]. In particular, the RF parameterization always predicts non-negative surface precipitation (Supplementary Figure 3). Similarly, in the remainder of this section we show that the RF parameterization conserves energy in the absence of external forcing (i.e., in the absence of radiative heating and surface fluxes of h_{L}).

RF-diff automatically respects energy conservation in the absence of external forcing since it predicts the turbulent diffusivity rather than the diffusive tendencies. To check energy conservation for RF-tend, we integrate the evolution equation for h_{L} (equation 1 in the methods section) in the vertical with density weighting, and then consider the contributions to the resulting equation that come from RF-tend (denoted with a superscript subgrid) to give an energy-conservation residual:

$$\text{residual} = \int_0^{\infty} \rho_0 \left(\frac{\partial h_{\mathrm{L}}}{\partial t}\right)_{\text{no}-\text{rad}}^{\text{subgrid}} dz + \overline{L}_p P_{\text{tot}}^{\text{subgrid}}(z=0) + \overline{L}_n S^{\,\text{subgrid}}(z=0). \quad \text{(S1)}$$

Here $\left(\frac{\partial h_{\mathrm{L}}}{\partial t}\right)_{\text{no}-\text{rad}}^{\text{subgrid}}$ is the subgrid tendency of h_{L} but excluding the contribution from radiative heating which is an external forcing. This tendency was evaluated by training a new RF-tend that predicts the radiative heating tendency and the sum of other tendencies

of h_{L} as separate outputs. This RF-tend performed similarly to our default RF-tend in all other regards. In deriving equation S1, we have neglected subgrid correlations between L_{p} and P_{tot} and between L_n and S, and as a result the residual will not be exactly zero even for the true subgrid tendencies. In addition, in evaluating the residual, the column energy change due to subgrid surface precipitation and sedimentation ($\overline{L}_p P_{\mathrm{tot}}^{\mathrm{subgrid}}(z = 0) + \overline{L}_n S^{\mathrm{subgrid}}(z = 0)$) was approximated to be $\overline{L}_p(P_{\mathrm{tot}}^{\mathrm{subgrid}}(z = 0) + S^{\mathrm{subgrid}}(z = 0))$ so that we could evaluate it using equation 7 in the methods section. This approximation leads to a small error to the extent that there is surface sedimentation.

The distribution of the energy-conservation residual for the true subgrid tendencies is shown in Supplementary Figure 2a and for the RF-predicted subgrid tendencies in Supplementary Figure 2b. In general, the residuals are very small, and the distribution of the residuals is similar for the true subgrid tendencies and the RF-predicted subgrid tendencies. The difference between the true and the RF-predicted residuals for each column was also calculated, and its distribution is shown in Supplementary Figure 2c.

Supplementary Figure 2b demonstrates that the RF parameterization respects energy conservation to a high degree of accuracy (less than 2% of the data has residuals that are larger in amplitude than $1\mathrm{W\ m^{-2}}$). The root mean square error in energy conservation is $0.35\mathrm{W\ m^{-2}}$ which is much smaller than the root mean square value of

$64.76\mathrm{W\ m^{-2}}$ for the vertical integral of the predicted energy tendencies. The mean bias error is $0.11\mathrm{W\ m^{-2}}$. We note that a similar mean bias error is found in the calculation of the energy-conservation residual from the true subgrid tendencies, and both are likely a result of the approximations we used in the calculation of the energy conservation residual rather than a violation of energy conservation (the mean bias error found in Supplementary Figure 2c is $0.0001\mathrm{W\ m^{-2}}$). The root mean square error of $0.35\mathrm{W\ m^{-2}}$ is substantially smaller than a reported value of $92\mathrm{W\ m^{-2}}$ in a previous study that used a NN to learn from a quasi-global simulation[2] with the caveat that the metric of errors in energy conservation in that study also included errors in predicted radiative heating and surface fluxes. We note also that energy conservation for a NN parameterization can be enforced by including it as a constraint in the NN architecture[3].

Supplementary Note 2

Here we describe an alternative RF parameterization approach in which q_{p} is not used as a variable. This alternative RF parameterization leads to stable simulations when implemented in SAM, and it gives similarly accurate results to the default approach for mean precipitation, but less accurate results for extreme precipitation in midlatitudes (Supplementary Figure 4). Since the alternative RF parameterization does not take q_{p} as an input, SAM in this case does not include q_{p} as a prognostic variable. Such a parameterization could be potentially very useful since q_{p} is a variable that changes on short time scales and therefore limits the size of the time step at coarse resolution. Furthermore, many climate models do not use q_{p} as a prognostic variable, and using an ML parameterization in these models requires a parameterization that does not use q_{p} as an input. Note that we haven't yet tested this parameterization with much larger time steps because turbulent diffusion as implemented in SAM also limits the time step.

The equations for the prognostic water and energy variables in SAM are described in equations 1-3 in the methods section. We define a new prognostic energy variable (H_{L}) that does not include the precipitating water (q_{p}):

$$H_{\mathrm{L}} = c_{\mathrm{p}}T + gz - L_{\mathrm{c}}q_c - L_{\mathrm{s}}q_{\mathrm{i}}. \tag{S2}$$

This can be written in terms of the original energy variable h_L as

$$H_\mathrm{L} = h_\mathrm{L} + L_\mathrm{p} q_\mathrm{p} \tag{S3}$$

where, $L_\mathrm{p} = L_\mathrm{c} + L_\mathrm{f}(1 - \omega_\mathrm{p})$ and ω_p is the partition function for precipitation which depends only on temperature in SAM[4]. In the following, we account for vertical variations of L_p in the vertical but neglect the smaller variations in the horizontal and in time. Taking the derivative with respect to time of equation S3 gives

$$\frac{\partial H_\mathrm{L}}{\partial t} = \frac{\partial h_\mathrm{L}}{\partial t} + L_\mathrm{p} \frac{\partial q_\mathrm{p}}{\partial t}. \tag{S4}$$

Substituting equations 1 and 3 from the methods section into equation S4, we get a prognostic equation for H_L:

$$\begin{aligned}\frac{\partial H_\mathrm{L}}{\partial t} = &-\frac{1}{\rho_0}\frac{\partial}{\partial x_i}(\rho_0 u_i H_\mathrm{L}) - \frac{1}{\rho_0}\frac{\partial}{\partial z}(L_n S) + L_\mathrm{p}\left(\frac{\partial q_\mathrm{p}}{\partial t}\right)_\mathrm{mic} + \left(\frac{\partial h_\mathrm{L}}{\partial t}\right)_\mathrm{rad} \\ &- \frac{1}{\rho_0}\frac{\partial F_{H_\mathrm{L} i}}{\partial x_i} + \frac{1}{\rho_0}\frac{\partial L_\mathrm{p}}{\partial z}(\rho_0 w q_\mathrm{p} + F_{q_\mathrm{p} z} - P_\mathrm{tot})\end{aligned} \tag{S5}$$

where $F_{H_\mathrm{L} i} = F_{h_\mathrm{L} i} + L_\mathrm{p} F_{q_\mathrm{p} i}$ and the last term on the right hand side results from heating from phase changes of precipitation.

Our aim is to make a parameterization for coarse-resolution simulations that does not include q_p. Therefore we assume that at coarse resolution we can neglect the horizontal fluxes of q_p and the time derivative of q_p in equation 3 from the methods section.

Integrating this equation vertically over the column and neglecting surface diffusive fluxes of $q_{\rm p}$ then gives an expression for the surface precipitation rate:

$$P_{\rm tot}(z=0) = -\int_0^\infty \left(\frac{\partial q_{\rm p}}{\partial t}\right)_{\rm mic} dz. \tag{S6}$$

The RF parameterization without $q_{\rm p}$ is similar in most respects to the RF parameterization with $q_{\rm p}$, but some changes are needed. First, RF-tend does not use $q_{\rm p}$ as a feature or predict its tendency as an output, and it predicts the tendency of $H_{\rm L}$ rather than the tendency of $h_{\rm L}$. Thus, the features for RF-tend are $X = (T, q_{\rm T}, |y|)$, and the outputs are $y = (H_{\rm L}^{\rm subg-tend}, q_{\rm T}^{\rm subg-tend})$. RF-diff is changed to predict the subgrid surface flux of $H_{\rm L}$ instead of $h_{\rm L}$. Second, RF-tend in this version predicts for $H_{\rm L}^{\rm subg-tend}$ the subgrid vertical advection and subgrid sedimentation terms added to the total value of $L_{\rm p}\left(\frac{\partial q_{\rm p}}{\partial t}\right)_{\rm mic} + \left(\frac{\partial h_{\rm L}}{\partial t}\right)_{\rm rad} + \frac{1}{\rho_0}\frac{\partial L_{\rm p}}{\partial z}(\rho_0 w q_{\rm p} + F_{q_{\rm p}z} - P_{\rm tot})$ in equation S5. Third, we do not apply the RF tendency of $q_{\rm T}$ due to subgrid vertical advection and sedimentation above $11.8{\rm km}$ to avoid a feedback that lead to a severe change in the global circulation. (This is likely to be a similar issue to an instability that occurred in a previous study on ML parameterization that also did not use $q_{\rm p}$ as a prognotic variable and in which this instability was dealt with by not including certain upper-level variables as features[5].) To avoid over-fitting the results presented here, we chose the same upper-level cutoff for these $q_{\rm T}$ tendencies ($11.8{\rm km}$) as was also used for radiative heating. We tested different upper-level

cutoffs ($11\mathrm{km}$, $9.5\mathrm{km}$) and different combinations of cutoff levels (different cutoff levels for each process) and found that all these choices led to simulations with qualitatively similar results.

When implementing the alternative RF parameterization in SAM, we remove q_p as a prognostic variable and change from h_L to H_L as a prognostic variable. We diagnose surface precipitation using equation S6 (plus any surface sedimentation). The approximations used in deriving equation S6 can result in negative instantaneous surface precipitation in rare cases. However, the surface precipitation averaged over 3 hours in the SAM simulations with this RF parameterization is negative less than 1% of the time and the negative values are smaller in magnitude than $0.2\mathrm{mm\ day}^{-1}$.

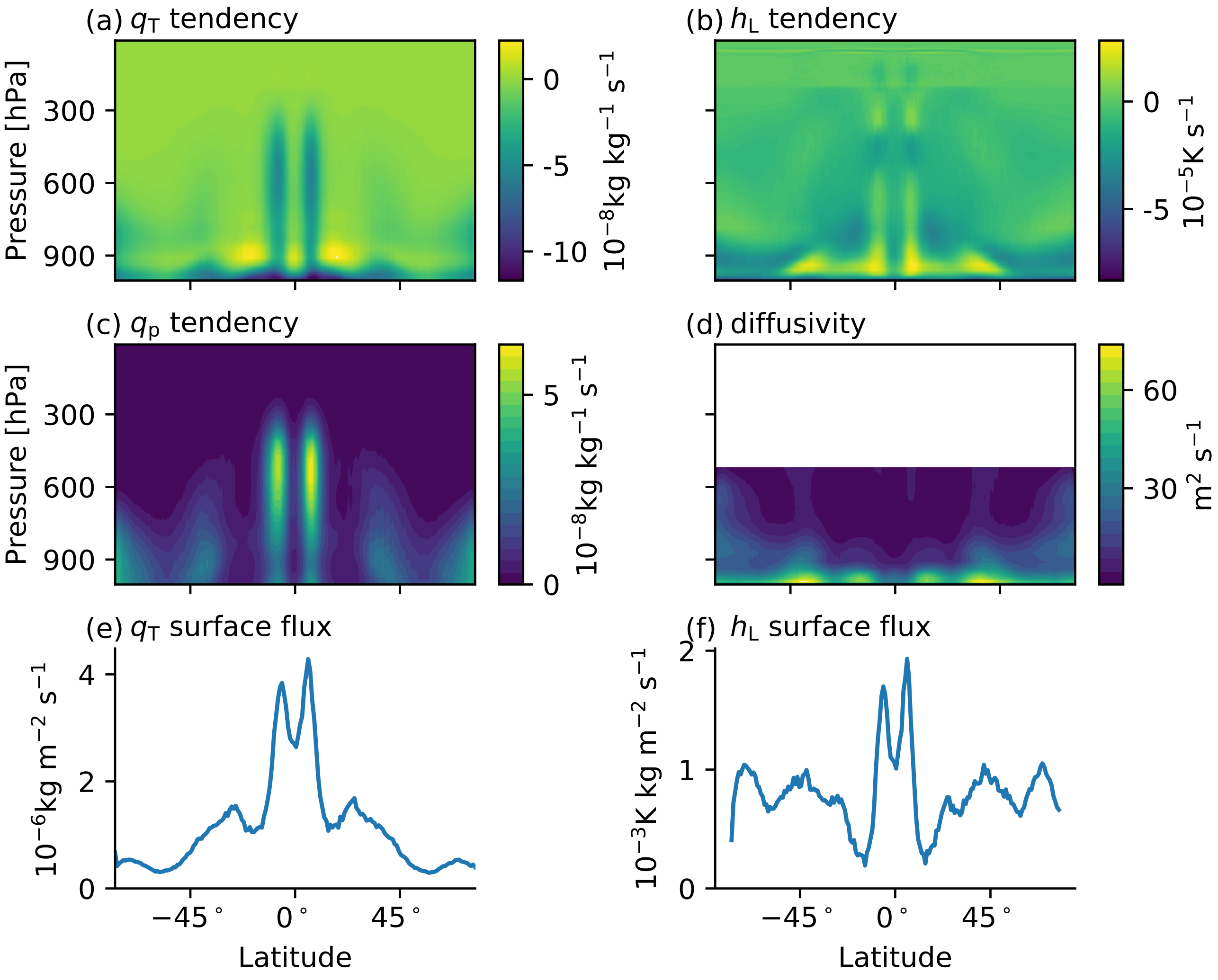

Supplementary Figure 1: **Mean true outputs of random forest parameterization.** The time- and zonal-mean for different true outputs of the random forest parameterization at x8: (a) subgrid tendency of q_{T}, (b) subgrid tendency of h_{L}, (c) subgrid tendency of q_{p}, (d) $\overline{D}$, (e) subgrid surface flux of q_{T}, and (f) subgrid surface flux of h_{L}.

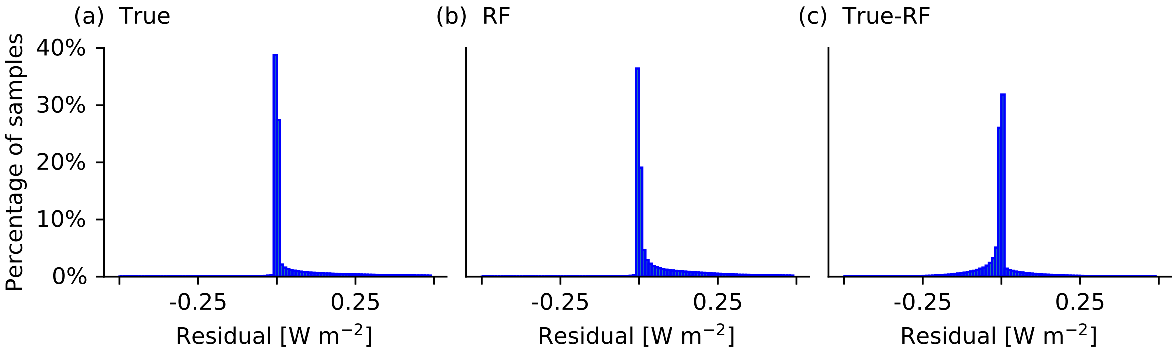

Supplementary Figure 2: **Distribution of energy-conservation residuals.** Equation S1 is applied to samples in the test dataset at x8 for the (a) true subgrid tendencies, (b) subgrid tendencies predicted by the random forest parameterization, and (c) the difference between the true and predicted subgrid tendencies. The bin size is $0.01\mathrm{Wm}^{-2}$.

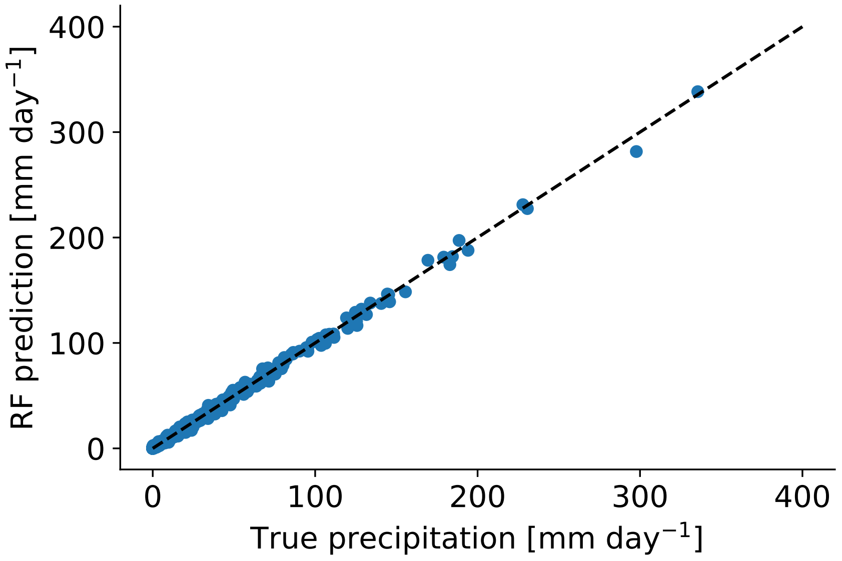

Supplementary Figure 3: **Offline performance for surface precipitation.** Scatter plot of true instantaneous surface precipitation coarse-grained to x8 versus the random forest (RF) prediction. The RF-predicted precipitation is calculated as the sum of the resolved precipitation and the subgrid correction. A random subset of 10,000 samples from the test set are shown for clarity. The black dashed line is the one-to-one line. We verified that the RF prediction gives non-negative precipitation values for all the $972,360$ test samples.

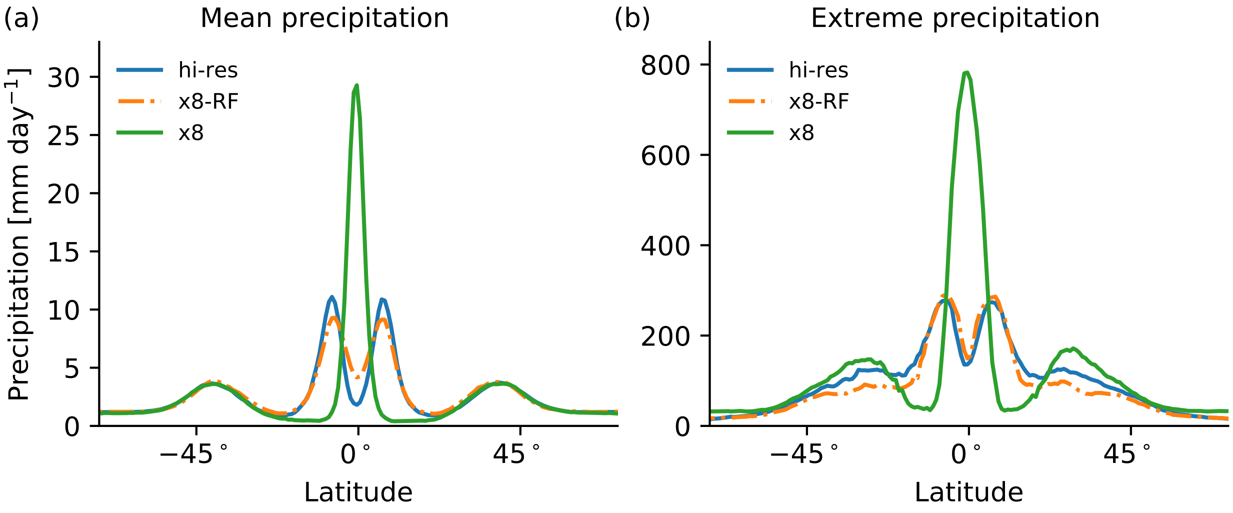

Supplementary Figure 4: **Mean and extreme precipitation in simulation with the alternative random forest-parameterization.** The simulation with the alternative random forest parameterization (Supplementary Note 2) does not use the precipitating water (q_p) as a variable. Shown are (a) mean precipitation and (b) 99.9th percentile of 3-hour precipitation at each latitude from the hi-res simulation (blue), x8-RF simulation without q_p (orange), and x8 simulation (green).

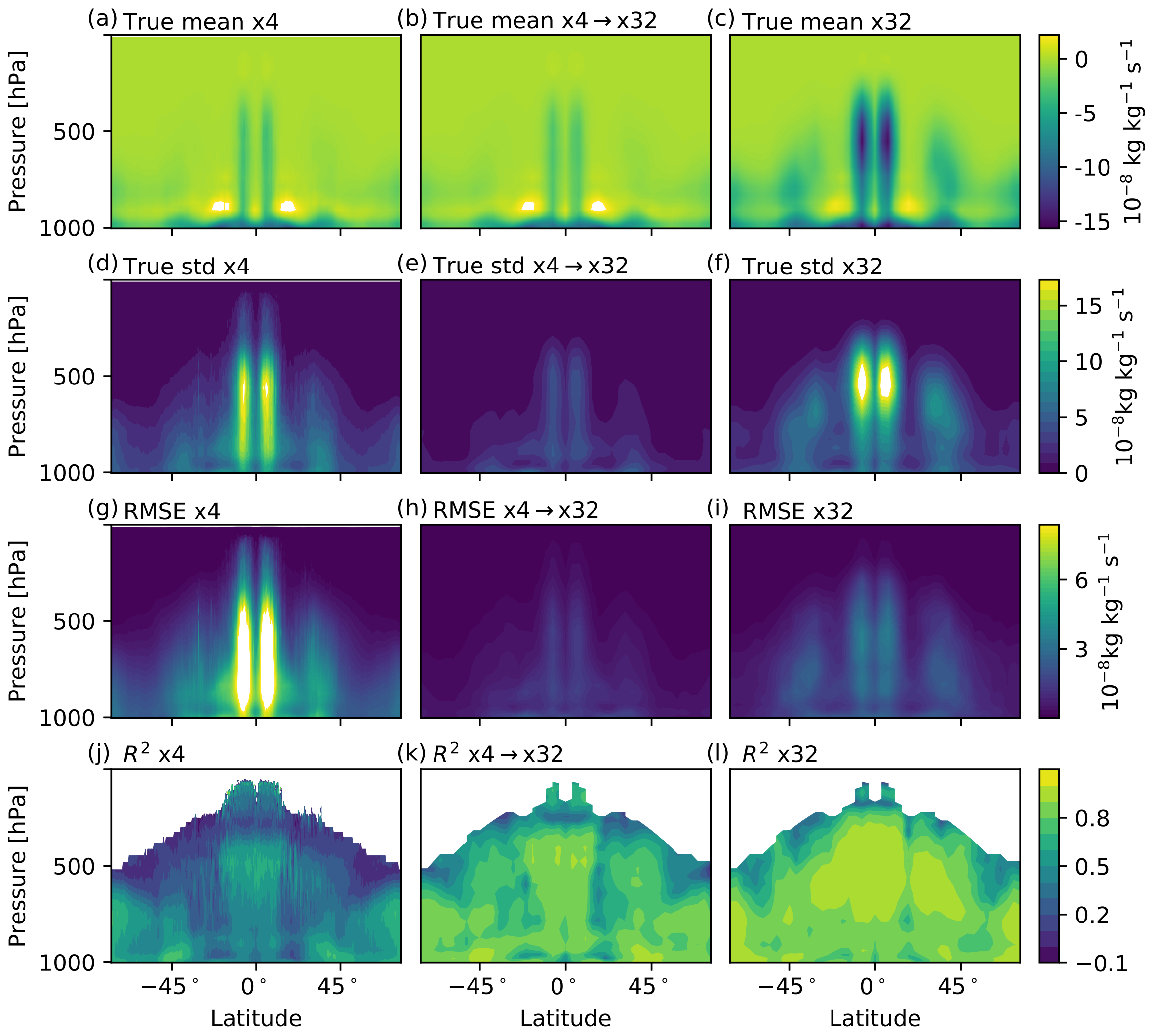

Supplementary Figure 5: **Offline comparison of parameterizations at a common grid spacing.** Offline results for the subgrid tendency of q_T: (a-c) true mean, (d-f) true standard deviation, (g-i) root mean square error for the random forest (RF) prediction, and (j-l) coefficient of determination (R^2) for the RF prediction. Results are shown for x4 (a,d,g,j), the subgrid tendency calculated and predicted at x4 and then coarse-grained to x32 (b,e,h,k), and x32 (c,f,i,l). Results shown in this figure are based on the alternative test dataset (see methods). Colorbar is saturated in panels f and g.

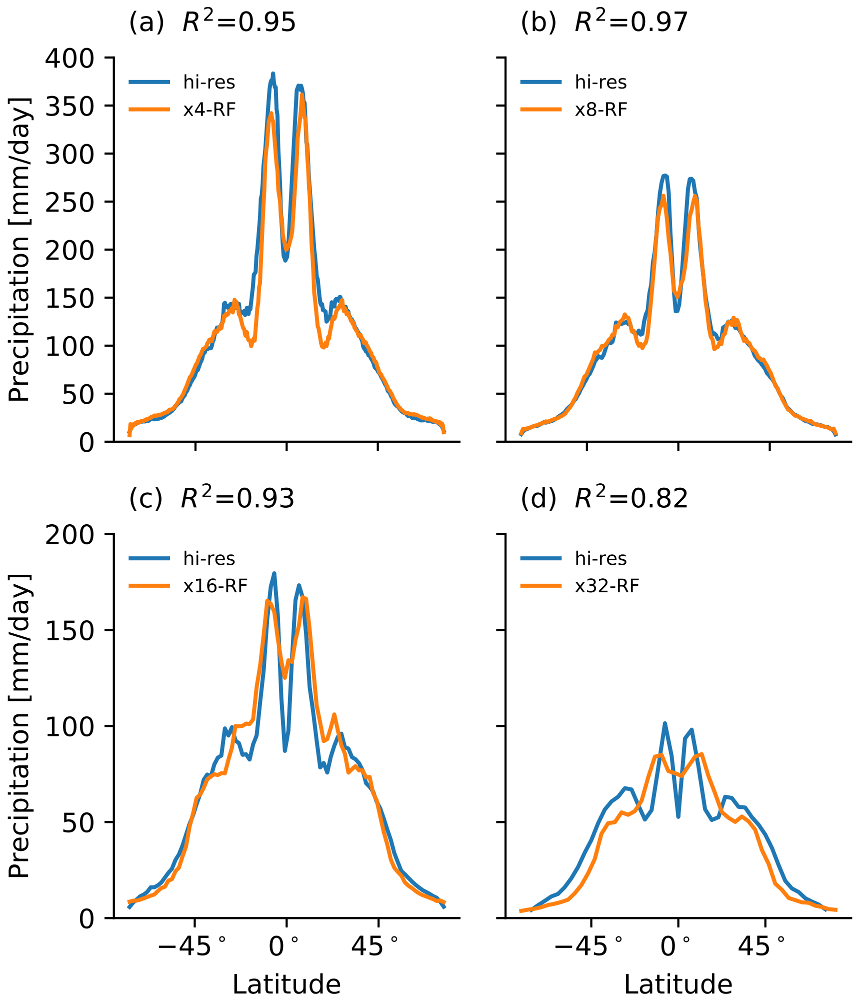

Supplementary Figure 6: **Extreme precipitation for simulations with different horizontal grid spacing.** Extreme precipitation as a function of latitude as measured by the 99.9th percentile of 3-hourly precipitation for: (a) x4, (b) x8, (c) x16 and (d) x32. Results are shown for the hi-res simulation (blue) and the coarse-resolution simulation with the random forest parameterization (orange). The precipitation rates for the hi-res simulation have been coarse-grained to the appropriate grid spacing prior to calculating the percentiles[6]. The coefficient of determination for each of the grid spacings is given above each panel.

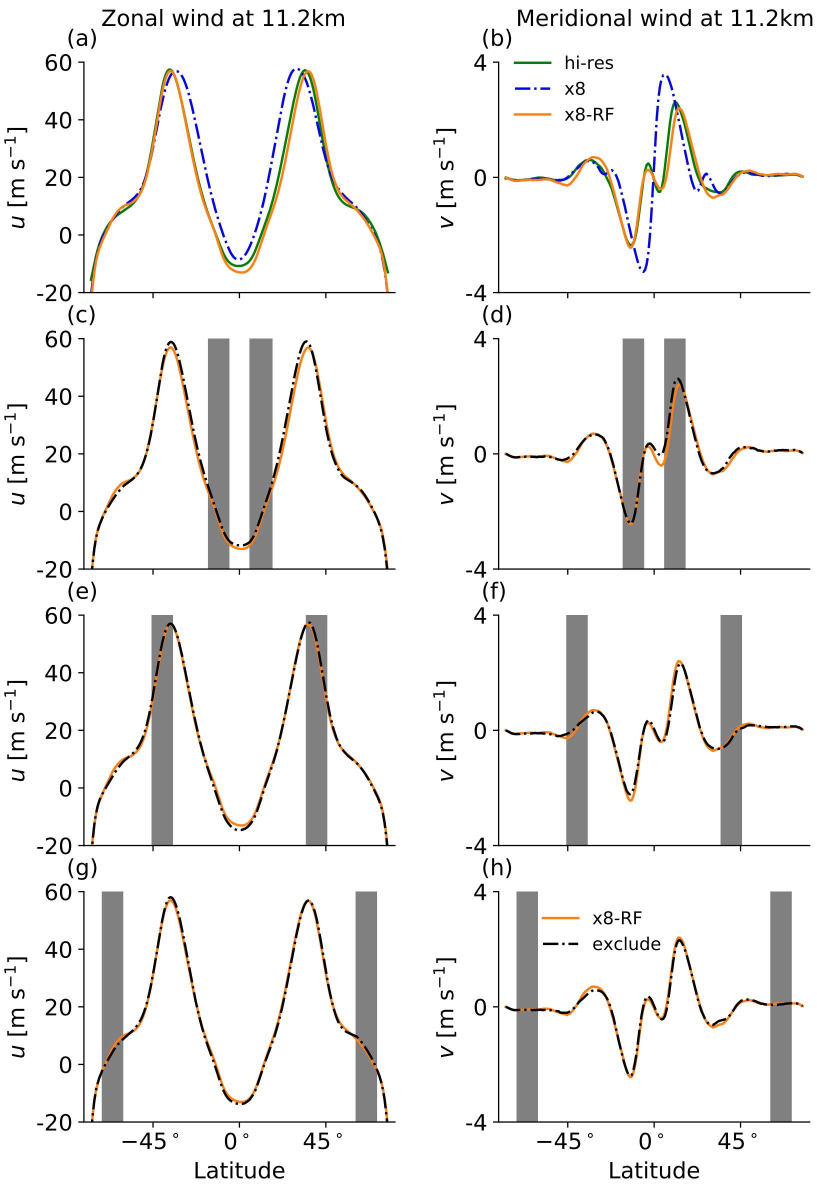

Supplementary Figure 7: **Online performance when excluding latitude bands during the training process.** Zonal- and time-mean of (a, c, e, g) the zonal wind at 11.2km and (b, d, f, h) the meridional wind at 11.2km. (a,b) The hi-res (green) and x8 (blue dash-dotted) simulations. (c-h) Simulations with x8-RFs (black dash-dotted) in which the training process excludes in both hemispheres the latitude bands (c,d) $5.1° - 15.5°$, (e,f) $34.5° - 44.9°$ and (g,h) $60.5° - 70.8°$. For comparison the results for x8-RF without any latitudes excluded in training are plotted in orange in all panels. Grey bars indicate latitude bands that where excluded during the training.

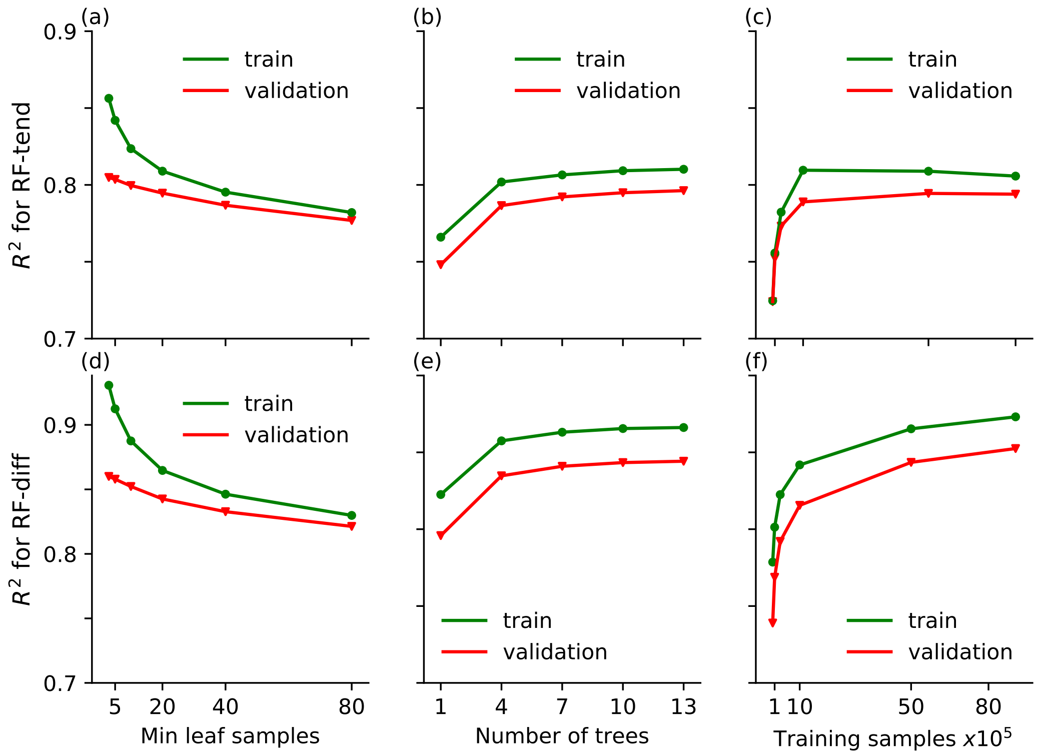

Supplementary Figure 8: **Hyperparameter tuning for random forests.** Coefficient of determination (R^2) for RF-tend (panels a-c, R^2 calculated for $q_{\mathrm{T}}^{\mathrm{subg-tend}}$) and RF-diff (d-f, R^2 calculated for diffusivity) as evaluated on the training dataset (green) and validation dataset (red) for x8 and different hyperparameter values: (a,d) minimum samples in each leaf, (b,e) number of trees in the forest, and (c,f) number of training samples. The hyperparameters that are used for both random forests (RFs) when implemented in SAM are 20 minimum samples in each leaf, 10 trees in the forest and $5,000,000$ training samples for the x4, x8, and x16 simulations. The hyperparameters that are used for both RFs in the x32 simulations are 7 minimum samples in each leaf, 10 trees in the forest and $1,969,020$ training samples.

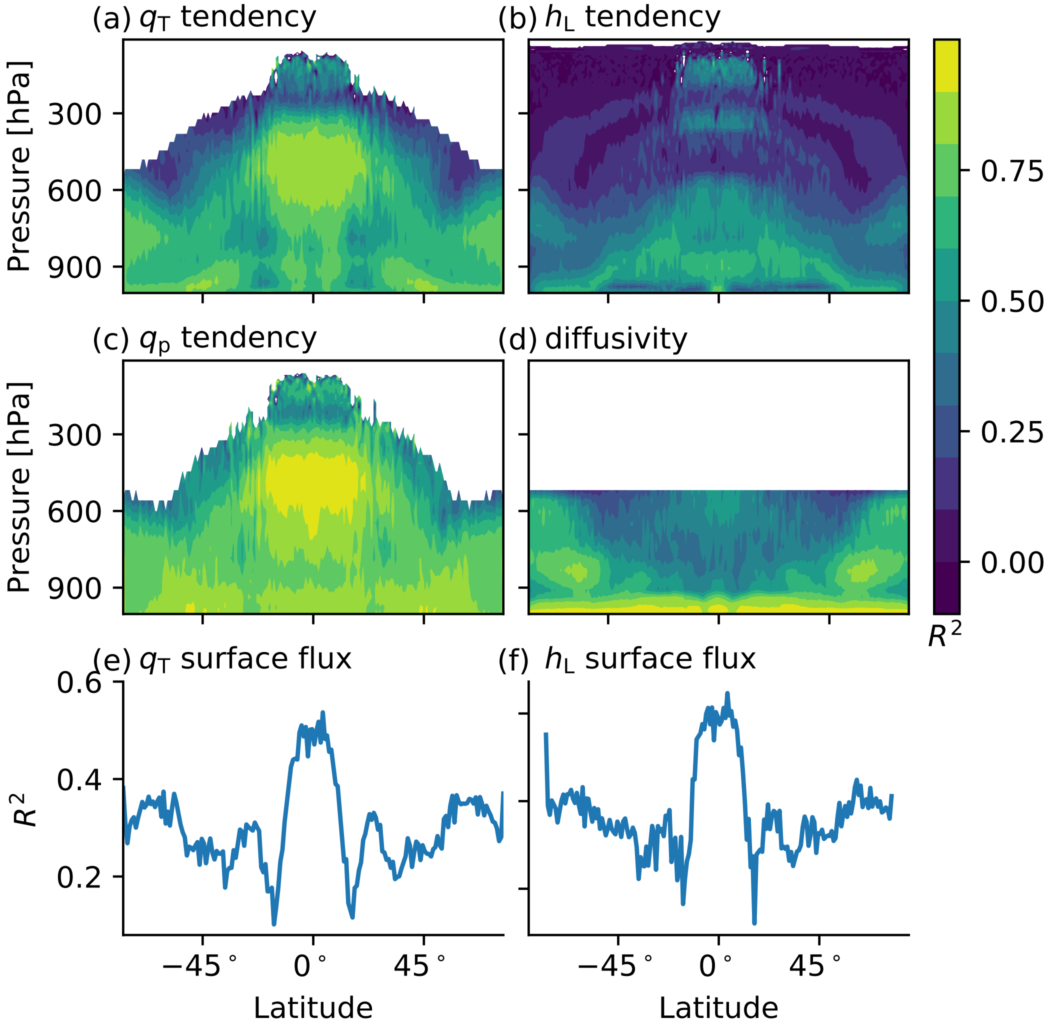

Supplementary Figure 9: **Offline performance of random forest parameterization.** The offline performance measured by the coefficient of determination (R^2) at x8 for: (a) subgrid tendency of q_T, (b) subgrid tendency of h_L, (c) subgrid tendency of q_p, (d) $\overline{D}$, (e) subgrid surface flux of q_T, and (f) subgrid surface flux of h_L. Results are based on the samples from the test dataset. R^2 is only shown where the variance is at least 0.1% of the mean variance over all latitudes and levels.

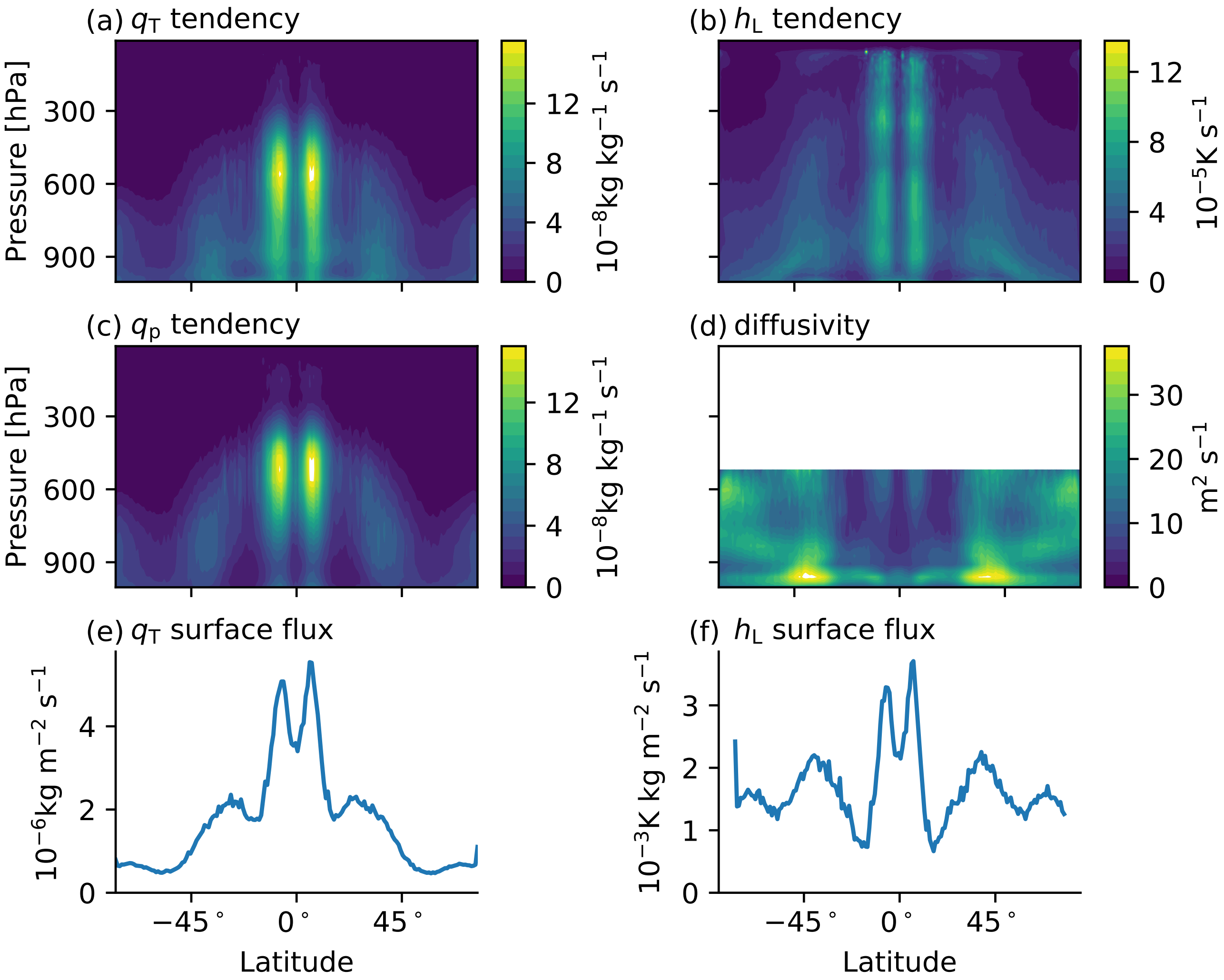

Supplementary Figure 10: **Standard deviation of true outputs for random forest parameterization.** The standard deviation of true outputs at x8: (a) subgrid tendency of q_{T}, (b) subgrid tendency of h_{L}, (c) subgrid tendency of q_{p}, (d) $\overline{D}$, (e) subgrid surface flux of q_{T}, and (f) subgrid surface flux of h_{L}.

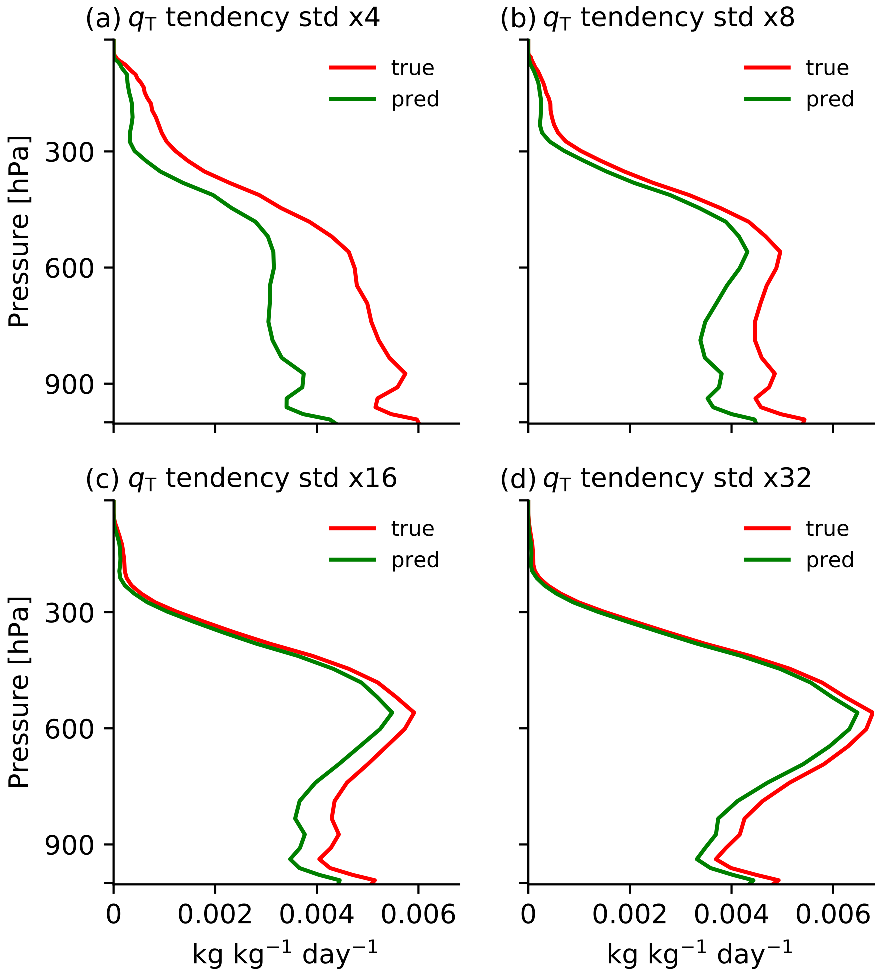

Supplementary Figure 11: **Vertical profiles of the standard deviation of outputs for different coarse graining factors.** The standard deviation of true (red) and random forest-predicted (green) subgrid tendency of q_T for different coarse-graining factors: (a) x4, (b) x8, (c) x16, and (d) x32. Results are evaluated based on the test dataset.

	x8-RF	x8
Eddy kinetic energy ($\mathrm{m}^2\ \mathrm{s}^{-2}$)	26.3 (0.97)	54.5 (0.88)
Zonal wind ($\mathrm{m\ s}^{-1}$)	2.2 (0.98)	4.5 (0.87)
Meridional wind ($\mathrm{m\ s}^{-1}$)	0.2 (0.87)	0.6 (-0.01)
Non-precipitating water ($\mathrm{g\ kg}^{-1}$)	0.1 (0.99)	0.4 (0.97)

Supplementary Table 1: **Online performance with and without random forest parameterization.** Online performance as measured by root mean square error (R^2 in parenthesis) of zonal- and time-mean variables for the coarse-resolution simulations with the random forest parameterization (x8-RF) and without the random forest parameterization (x8) as compared to the target hi-res simulation. The eddy kinetic energy is defined with respect to the zonal and time mean.

	$q_{\mathrm{T}}^{\mathrm{subg-tend}}$	$h_{\mathrm{L}}^{\mathrm{subg-tend}}$	$q_{\mathrm{p}}^{\mathrm{subg-tend}}$	$\overline{D}$	$q_{\mathrm{T}}^{\mathrm{surf-flux}}$	$h_{\mathrm{L}}^{\mathrm{surf-flux}}$
x4	0.56	0.31	0.73	0.72	0.30	0.29
x8	0.80	0.48	0.88	0.84	0.48	0.44
x8-no-$\|y\|$	0.79	0.48	0.88	0.84	0.47	0.42
x16	0.90	0.64	0.93	0.93	0.60	0.57
x32	0.95	0.75	0.96	0.96	0.78	0.76

Supplementary Table 2: **Offline performance of random forest parameterizations as measured by R^2.** The offline performance is given for different coarse-graining factors and different outputs of the random forests. For x8-no-$|y|$, the distance from equator was not used as a feature. For the tendencies and turbulent diffusivity, all levels used are included when calculating R^2. All results are based on the test dataset.

	$q_{\mathrm{T}}^{\mathrm{subg-tend}}$	$h_{\mathrm{L}}^{\mathrm{subg-tend}}$	$q_{\mathrm{p}}^{\mathrm{subg-tend}}$	$\overline{D}$	$q_{\mathrm{T}}^{\mathrm{surf-flux}}$	$h_{\mathrm{L}}^{\mathrm{surf-flux}}$
	$10^{-8}\times$	$10^{-5}\times$	$10^{-8}\times$		$10^{-6}\times$	$10^{-3}\times$
	kg kg^{-1}s^{-1}	K s^{-1}	kg kg^{-1} s^{-1}	m^2 s^{-1}	kg m^{-2} s^{-1}	K kg m^{-2} s^{-1}
x4	2.50	3.65	1.23	15.27	1.76	1.36
x8	1.64	2.27	0.94	9.88	1.68	1.35
x8-no-$\|y\|$	1.65	2.28	0.94	10.02	1.69	1.37
x16	1.22	1.54	0.83	6.64	1.76	1.52
x32	0.94	1.10	0.72	4.28	1.69	1.58

Supplementary Table 3: **Offline performance of random forest parameterizations as measured by root mean square error**. The offline performance is given for different coarse-graining factors and different outputs of the random forests. For x8-no-$|y|$, the distance from equator was not used as a feature. For the tendencies and turbulent diffusivity, all levels used are included when calculating root mean square error. All results are based on the test dataset.

	$q_{\mathrm{T}}^{\mathrm{subg-tend}}$	$h_{\mathrm{L}}^{\mathrm{subg-tend}}$	$q_{\mathrm{p}}^{\mathrm{subg-tend}}$
	10^{-8} kg kg^{-1}s^{-1}	10^{-5} K s^{-1}	10^{-8} kg kg^{-1} s^{-1}
x4	2.57 (0.55)	3.79 (0.29)	1.28 (0.71)
x4→x32	0.49 (0.93)	0.39 (0.82)	0.21 (0.95)
x32	0.97 (0.95)	1.16 (0.73)	0.74 (0.96)

Supplementary Table 4: **Offline comparison of random forest parameterizations at a common grid spacing.** Offline performance as measured by root mean square error (R^2 values in brackets) for different outputs of RF-tend for x4, coarse graining of the subgrid tendencies calculated and predicted at x4 to x32 grid spacing (x4→x32), and x32. Results in this table are based on the alternative test dataset (see methods).

Supplementary References

1. O'Gorman, P. A. & Dwyer, J. G. Using machine learning to parameterize moist convection: Potential for modeling of climate, climate change, and extreme events. *J. Adv. Model. Earth Sys.* **10**, 2548–2563 (2018).

2. Brenowitz, N. D. & Bretherton, C. S. Prognostic validation of a neural network unified physics parameterization. *Geophys. Res. Lett.* **45**, 6289–6298 (2018).

3. Beucler, T., Rasp, S., Pritchard, M. & Gentine, P. Achieving conservation of energy in neural network emulators for climate modeling. *arXiv preprint arXiv:1906.06622* (2019).

4. Khairoutdinov, M. F. & Randall, D. A. Cloud resolving modeling of the ARM summer 1997 IOP: Model formulation, results, uncertainties, and sensitivities. *J. Atmos. Sci.* **60**, 607–625 (2003).

5. Brenowitz, N. D. & Bretherton, C. S. Spatially extended tests of a neural network parametrization trained by coarse-graining. *J. Adv. Model. Earth Sys.* **11**, 2727–2744 (2019).

6. Chen, C.-T. & Knutson, T. On the verification and comparison of extreme rainfall indices from climate models. *J. Climate* **21**, 1605–1621 (2008).
